# Assessment of Machine Learning Detection of Environmental Enteropathy and Celiac Disease in Children

**DOI:** 10.1001/jamanetworkopen.2019.5822

**Published:** 2019-06-14

**Authors:** Sana Syed, Mohammad Al-Boni, Marium N. Khan, Kamran Sadiq, Najeeha T. Iqbal, Christopher A. Moskaluk, Paul Kelly, Beatrice Amadi, S. Asad Ali, Sean R. Moore, Donald E. Brown

**Affiliations:** 1 Division of Pediatric Gastroenterology and Hepatology, Department of Pediatrics, University of Virginia, Charlottesville; 2 Department of Pediatrics and Child Health, Aga Khan University, Karachi, Pakistan; 3 Systems and Information Engineering, University of Virginia, Charlottesville; 4 Deparment of Pathology, University of Virginia, Charlottesville; 5 Blizard Institute, Barts and The London School of Medicine, Queen Mary University of London, London, United Kingdom; 6 Tropical Gastroenterology and Nutrition Group, University of Zambia School of Medicine, Lusaka, Zambia; 7 Data Science Institute, University of Virginia, Charlottesville

## Abstract

**Importance:**

Duodenal biopsies from children with enteropathies associated with undernutrition, such as environmental enteropathy (EE) and celiac disease (CD), display significant histopathological overlap.

**Objective:**

To develop a convolutional neural network (CNN) to enhance the detection of pathologic morphological features in diseased vs healthy duodenal tissue.

**Design, Setting, and Participants:**

In this prospective diagnostic study, a CNN consisting of 4 convolutions, 1 fully connected layer, and 1 softmax layer was trained on duodenal biopsy images. Data were provided by 3 sites: Aga Khan University Hospital, Karachi, Pakistan; University Teaching Hospital, Lusaka, Zambia; and University of Virginia, Charlottesville. Duodenal biopsy slides from 102 children (10 with EE from Aga Khan University Hospital, 16 with EE from University Teaching Hospital, 34 with CD from University of Virginia, and 42 with no disease from University of Virginia) were converted into 3118 images. The CNN was designed and analyzed at the University of Virginia. The data were collected, prepared, and analyzed between November 2017 and February 2018.

**Main Outcomes and Measures:**

Classification accuracy of the CNN per image and per case and incorrect classification rate identified by aggregated 10-fold cross-validation confusion/error matrices of CNN models.

**Results:**

Overall, 102 children participated in this study, with a median (interquartile range) age of 31.0 (20.3-75.5) months and a roughly equal sex distribution, with 53 boys (51.9%). The model demonstrated 93.4% case-detection accuracy and had a false-negative rate of 2.4%. Confusion metrics indicated most incorrect classifications were between patients with CD and healthy patients. Feature map activations were visualized and learned distinctive patterns, including microlevel features in duodenal tissues, such as alterations in secretory cell populations.

**Conclusions and Relevance:**

A machine learning–based histopathological analysis model demonstrating 93.4% classification accuracy was developed for identifying and differentiating between duodenal biopsies from children with EE and CD. The combination of the CNN with a deconvolutional network enabled feature recognition and highlighted secretory cells’ role in the model’s ability to differentiate between these histologically similar diseases.

## Key Points

**Question:**

Can deep learning image analysis distinguish pathological vs healthy features in duodenal tissue?

**Findings:**

In this diagnostic study, a deep learning convolutional neural network was trained on 3118 images from duodenal biopsies of patients with environmental enteropathy, celiac disease, and no disease. The convolutional neural network achieved 93.4% case-detection accuracy, with a false-negative rate of 2.4%, and automatically learned microlevel features in duodenal tissue, such as alterations in secretory cell populations.

**Meaning:**

A machine learning–based feature detection platform distinguished pathological from healthy tissue in gastrointestinal biopsy images.

## Teaser

This diagnostic study assesses a convolutional neural network to enhance the detection of pathologic morphological features in duodenal tissue among children with environmental enteropathy, celiac disease, and no disease.

## Introduction

The interpretation of clinical biopsy images for disease diagnoses can be challenging when clinicians are faced with distinguishing between distinct but related conditions. Recently, increasing attention has been paid to methods in artificial intelligence that help clinicians to translate big data (ie, biomedical images and patient biosample data) into accurate and quantitative diagnostics.^1^ To our knowledge, most computer modeling enhancements in health care, particularly in image analysis, have focused on feature engineering, ie, asking a computer to evaluate prespecified, explicit image features to permit computational algorithms to detect disease or specified lesions. In contrast, deep learning or a convolutional neural network (CNN) is a form of artificial intelligence that includes machine learning techniques designed to process data and interpret it (eg, by detecting and segmenting multiple pixel intensities within a single image and labeling features at a pixel-by-pixel level).^2,3^

Machine learning and its subtypes (eg, CNNs) are an extension of the traditional tools and methods of statistical analysis (eg, linear regression, comparative *t* tests).^4^ In 2017, Ehteshami Bejnordi et al^1^ demonstrated the use of deep learning algorithms to interpret whole-slide pathology images. They used annotated images of metastases in lymph node biopsies to train various algorithms and showed that some algorithms achieved better diagnostic performance compared with a panel of trained pathologists.^1^

We hypothesized that deep learning algorithms for pathology slide evaluation could recognize complex disease phenotypes that cannot be measured via molecular approaches and are dependent on tissue diagnostics. We wanted to develop diagnostic methods to enable us to correlate patient-level numerical metadata, including biomarkers, with invasively obtained data (eg, tissue biopsies). Our specific focus was on pediatric undernutrition, which is estimated to cause approximately 45% of the 5 million deaths annually in children younger than 5 years worldwide.^5^ There are many manifestations of early childhood undernutrition; stunting (linear growth failure, length-for-age *z* score <−2) is among the most common, affecting approximately 155 million children younger than 5 years.^6^ Stunting is a clinical marker for devastating, sometimes irreversible, deficiencies, which have adverse cognitive, physical, immunologic, and socioeconomic effects.^7,8,9,10^

A common cause of stunting in the United States is celiac disease (CD), with an estimated 1% prevalence.^11^ Celiac disease is an immune-mediated, small-bowel enteropathy triggered by gluten sensitivity in people with genetic susceptibility.^12^ Environmental enteropathy (EE), another similar but distinct condition, is thought to be a key factor underlying stunting in children residing in low-income and middle-income countries.^13,14^ Environmental enteropathy is an acquired small-intestinal condition that is proposed to be a consequence of the continuous burden of immune stimulation by fecal-oral exposure to enteropathogens, leading to persistent, nonspecific chronic inflammation.^15,16,17^ Environmental enteropathy and CD have been described as overlapping enteropathies.^17,18,19,20,21^ Currently, the standard diagnostic criteria for these diseases is the evaluation of a small-intestinal biopsy obtained via an endoscopic procedure, which requires sedation.^17,22^ Between 4 and 6 biopsies are required for diagnosis,^23^ and because only parts of the bowel are affected in some cases, patients may require multiple endoscopic procedures. Therefore, there is a need to develop methods that allow feature extraction from biopsies of children with undernourishment and stunting for further analysis, ie, correlation with numerical metadata. This would aid traditional pathology-based diagnoses and pave the way for future gastrointestinal diagnostics that depend less on obtaining intestinal biopsies and more on noninvasive interpretations of intestinal histological features for both diagnosis and follow-up.

The pathologist’s interpretation of histological tissue is a unique skill set; pathologists associate specific clinical information with biopsy findings to suggest a presumptive diagnosis.^24^ However, it is difficult to translate this ability into quantifiable measurements that can be used for statistical analyses with other numerical metadata—most tissue measurements are subjective, whether they are morphometry, immunohistochemistry, or fluorescence intensity quantification.^25,26,27,28^

We propose a deep learning–based image analysis platform for the automated extraction of quantitative morphologic phenotypes from gastrointestinal biopsy images to identify novel features that could be used to help differentiate between overlapping conditions (EE and CD). Our overarching aim is to develop methods in data science to support the integration of this data with clinical and molecular data, enabling the construction of biologically informative and clinically useful integrative prognostic models for pediatric undernutrition. The primary aim of the present study is to build and deconstruct a deep learning network, using unannotated images of duodenal biopsy slides, which would characterize intestinal mucosal alterations and distinguish between EE, CD, and healthy tissue. We hypothesized that advances in deconvolutional neural networks (DNNs) could mimic the pathologist’s skill set, including the ability to identify novel features in understudied diseases (eg, EE). Deconvolutional neural networks construct hierarchical image representations that are top-down projections representing structures that have stimulated particular feature maps.^29,30^ They are hypothesized to be able to look for distinctive patterns in input images^29,30^ and, therefore, could find key distinguishing features between many overlapping diseases, not just EE and CD.

## Methods

This study is a prospective diagnostic study designed to develop and validate a predictive machine learning model for the interpretation of duodenal biopsy slides and feature detection in diseased vs healthy duodenal tissue. Its report follows the Transparent Reporting of a Multivariable Prediction Model for Individual Prognosis or Diagnosis (TRIPOD) reporting guideline.^31^ The data were collected, prepared, and analyzed from November 2017 to February 2018. This study was approved by the University of Virginia institutional review board (waiver of consent granted), the Ethical Review Committee of Aga Khan University in Karachi, Pakistan (informed consent obtained from parents and/or guardians), and the Biomedical Research Ethics Committee of the University of Zambia in Lusaka, Zambia (informed consent obtained from caregivers).

### Image Analysis Data Sets

We obtained 3118 segmented images from 121 hematoxylin-eosin (H-E)–stained duodenal biopsy glass slides from 102 patients, labeled as EE, CD, or control. Primary study physicians at each site made all diagnoses based on histological and clinical findings. Biopsy slides for patients with EE were obtained from the Aga Khan University Hospital (29 slides from 10 patients) and the University of Zambia Medical Center (16 slides from 16 patients). Biopsy slides for patients with CD (34 slides from 34 patients) and the control group (42 slides from 42 patients) were obtained from the Biorepository and Tissue Research Facility at the University of Virginia (eAppendix 1 in the Supplement).

### Biological Sample Collection

Data on various biomarkers obtained from blood, urine, and/or fecal samples of patients from Pakistan and Zambia with EE were also obtained. The complete details of the acquisition and handling of the specimens have been outlined in articles by Iqbal et al^32^ (for patients in Pakistan) and Amadi et al^33^ (for patients in Zambia). These biomarkers were used to propose an algorithmic framework to correlate this numerical metadata with biopsy features. Since limited and variable biomarkers were obtained in each of these studies, biological inferences could not be made from our results. Therefore, our primary goal was to develop a correlation algorithm to test in a larger, more comprehensive data set.

### Statistical Analysis

Descriptive statistics were performed using the R coding language and RStudio (The R Institute). Anthropometric measurements and age were used to calculate median and interquartile range *z* scores for length for age and height for age, weight for age, and weight for height. These were calculated using reference guidelines and R application macros available through the World Health Organization.^34,35^ Weight-for-age *z* scores were only calculated for children younger than 10 years because no reference guideline exists for patients older than 10 years owing to the discrepancies in height and body mass index because of pubertal changes.^35^

#### Convolutional Neural Network and DNN Framework

**Figure 1.**
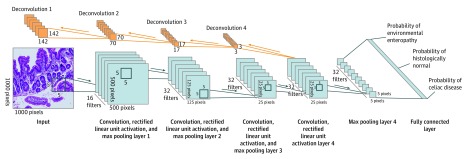
Illustration of Proposed Convolutional Neural Network Classification and Visualization Framework The convolutional neural network consists of 4 convolution layers and 1 fully connected layer. Each convolution layer consists of 3 sublayers: (1) a convolution layer, (2) a rectified linear unit activation layer, and (3) a max pooling layer. Deconvolution layers increase image resolution and find locations with high activations. The input image represents a hematoxylin-eosin–stained duodenal biopsy image (original magnification ×100).

We proposed a CNN, based on AlexNet,^36^ to perform multiclass classification on biopsy images (Figure 1). Importantly, while based on AlexNet,^36^ which requires the least amount of training data vs deeper architectures (eg, VGG-16^37^ and ResNet^38^), several variations to the architecture were made to reduce the number of trainable parameters to enable using a smaller data set. Variations included the use of (1) 1 convolution network pipeline vs 2, (2) 4 convolutions vs 5, and, most importantly, (3) 1 fully connected layer with 1024 neurons vs 2 fully connected layers with 4096 neurons. The network consisted of (1) 4 convolution layers, each followed by a rectified linear unit layer^39^ and a max pooling layer; (2) 1 fully connected layer; (3) 1 dropout layer; and (4) 1 softmax layer.

The 4 convolution layers had 16, 32, 32, and 32 feature maps, respectively, with pixel-by-pixel filter sizes of 5 × 5, 5 × 5, 5 × 5, and 3 × 3, respectively. Each convolution’s input layer was 0 padded, ensuring equal sizes of input and output. The max pooling layers’ window sizes were set to 2 × 2, 4 × 4, 5 × 5, and 5 × 5, respectively. The stride in convolution and max pooling layers was set to 1. Given an input of 1000 × 1000 × 3, the fourth max pooling layer would generate a 5 × 5 output for each feature map. The output of the 32 feature maps was flattened and concatenated before being connected to the fully connected layer. The dropout layer had a dropout probability of 0.5,^40^ and the softmax layer generated 3 probabilities: (1) likelihood of image being healthy duodenal tissue, (2) likelihood of image being duodenal tissue with CD, or (3) likelihood of image being duodenal tissue with EE.

An additional component was added to the network for high activation visualization, which is conceptually similar to a DNN.^29^ However, we increased the resolution starting from a low-resolution output layer, and instead of deconvoluting the entire output layer, only the highest activation for each feature map was traced back to the source image.

We trained 1 DNN model on the entire data set. Then, we visualized the patches with the highest activations from each of the 32 feature maps at layer 4 with respect to the different classes.

#### Image Processing

We had 2 image sources: (1) digitized whole-slide images from Pakistan (single biopsy per slide) and Zambia (multiple biopsies per slide) and (2) scanned slide images at magnification ×40 and ×100 of CD and control slides (multiple biopsies per slide). Both image formats had relatively high resolutions (ie, ranging from 2288 × 1356 pixels to 18 304 × 14 926 pixels).

However, although most discriminating features can only be observed at high resolution, it is impractical to input such high-resolution images into the network. Therefore, methods of artificial data augmentation, including segmentation, horizontal and vertical reflection of randomly selected patches, and γ correction, were used to produce a more practical input data set (eFigure 1 in the Supplement).

Each biopsy was segmented into multiple 1360 × 1024 images. During testing, averages from all images from each patient were used for the final prediction. To adjust for artifact and hue variations between slides, color augmentation experiments were conducted, including γ correction, contrast-limited adaptive histogram equalization, and γ correction with contrast-limited adaptive histogram equalization.^41^ A 10-fold cross-validation performance of the CNN found that the use of γ correction alone provided the best performance.^41^ Therefore, γ correction was applied to the data set with a random γ value of 0.5 to 2.0 to account for appreciable intersite color differences in H-E staining.

For each image, ten 1000 × 1000 patches and their horizontal and vertical reflections (for variability in feature orientation, such as villi, within the same and different images) were randomly selected. The size of our data set increased by a factor of 30 and enabled the algorithm to learn translation and rotation invariant features.

As a result of data augmentation, approximately 85 000 images were included in each fold (approximately 76 059 for training and 8541 for testing). For testing, 15 patches were generated from each image: 1 central patch, 4 corner patches, and their horizontal and vertical reflections. Then, the average probability of EE was calculated from these patches. The likelihood of EE in a biopsy was computed as the mean of its segments’ estimated probabilities.

#### Feature Prediction *t *Tests

Zeiler and Fergus^29^ and Zeiler et al^30^ suggested that filters from the CNN would look for distinctive patterns in input images. The degree to which a pattern match is found in the input image is reflected by the activation value, and a higher value corresponds to a better match. Therefore, image patches were run from the different classes, and the maximum activation value per CNN filter was collected. A *t* test was run to test the hypothesis that activation values from a class (eg, EE) are significantly higher than those generated by other classes (eg, CD or control); this test served as a proxy for testing the prevalence of various tissue patterns in different biopsies (eFigure 2 in the Supplement).

#### Lasso Regression Models for Biomarker Correlation

Lasso regression models were built to correlate EE biomarkers with activation maps and to predict EE biopsy features. The lasso model was chosen to add regularization to the regression to avoid overfitting and to promote sparsity in the feature space to reduce the number of input biomarkers used to predict biopsy features. In the EE studies from Pakistan and Zambia, each patient with EE was associated with various variables, including noninvasive biomarkers (from blood, urine, and stool) that described the patient’s clinical situation. Biomarkers have been used to detect EE and other gastrointestinal diseases, such as biliary atresia and inflammatory bowel disease.^42,43,44,45,46,47,48^ However, to our knowledge, there has been no work on estimating or synthesizing biopsies from biomarkers.

Only data from Pakistan were used for this biopsy-biomarker correlation framework, which consisted of 2 components: (1) CNN activations and (2) lasso regression (eFigure 3 in the Supplement). The correlation process involved 5 steps. First, for training, the CNN contained 32 different feature maps at the fourth layer; each feature map searched for a specific pixel pattern (ie, morphological feature) in the input images (derived from dividing biopsies into multiple images). Each image would produce 25 × 25 pixel convoluted values for each feature map. Second, a maximum operator function was performed on the activations across all the images per each biopsy. The maximum value of the final 625 convoluted values corresponded to a 142 × 142 pixel segment that maximally activated the feature map. Therefore, each biopsy was mapped onto 32 values that corresponded to 32 segments. Third, each case was associated with multiple variables. Thus, 32 lasso regression models were built to correlate variables with feature map activation values; 32 image segments of 142 × 142 pixels were extracted from each biopsy, and their activation values at layer 4 were correlated with biomarkers. Fourth, for testing, the trained lasso regression models estimated 32 activation values from the biomarkers. Rectified linear units generate nonnegative activations; therefore, response variables for the regression models only have positive values. However, because of the possibility that using the trained models for prediction may generate negative estimates, we removed negative estimates from the testing cases’ estimates. Fifth, the estimated values were used to obtain training image sections with similar activation values; these sections were used to reconstruct the testing image. The underlying assumption was that 2 regions from 2 images producing similar activation values would likely contain similar pixel patterns.

The correlation model was evaluated with 2 approaches. First, for each 10-fold cross-validation testing case, we estimated 32 activations from biomarkers using the trained lasso regression models. Then, we applied the CNN models to these cases and computed the mean squared error (MSE) between estimated and actual activations. Next, random forest models were used to estimate each biomarker’s importance in predicting biopsy features. We then tested the predictive power of the subsets of the most important biomarkers. Starting with a model using all biomarkers, we sequentially removed the least important feature, retrained the model, and estimated the cross-validation per-image and per-biopsy MSE. Second, we applied the trained CNN models on testing cases and obtained the 32 regions that corresponded to the highest activation values for the 32 feature maps.

## Results

### Background Clinical Characteristics of Patient Populations

Table 1 summarizes the participants’ background characteristics. The median (interquartile range) age of the 102 participants was 31.0 (20.3-75.5) months, and there was a roughly equal sex distribution, with 53 boys (51.9%). Overall, 26 patients (25.5%) were diagnosed as having EE, 34 patients (33.3%) were diagnosed as having CD, and 42 patients (41.2%) had healthy duodenal tissue. More characteristics are described in eAppendix 2 in the Supplement.

**Table 1.** Background Clinical Characteristics of Patient Population

Characteristic	No. (%)				
	Total Participants	Patients With Environmental Enteropathy		Patients With Celiac Disease, United States	Patients With No Disease, United States
		Pakistan	Zambia		
Diagnosis	102 (100)	10 (9.8)	16 (15.7)	34 (33.3)	42 (41.2)
Age, median (IQR), mo	31.0 (20.3 to 75.5)	22.0 (20.0 to 23.0)	16.5 (10.5 to 21.0)	129.0 (72.5 to 180.8)	31.5 (22.0 to 49.8)
Sex					
Boys	53 (51.9)	5 (50.0)	10 (62.5)	12 (35.0)	26 (62.0)
Girls	49 (48.1)	5 (50.0)	6 (37.5)	22 (65.0)	16 (38.0)
Images^a^	121 (100)	29 (24.0)	16 (13.2)	34 (28.0)	42 (34.7)
Weight-for-age *z* score, median (IQR)	−1.00 (−3.10 to 0.06)	−3.40 (−3.78 to −2.46)	−3.75 (−5.23 to −3.21)	−0.14 (−0.77 to 0.24)^b^^,^^c^	−0.36 (−1.28 to 0.93)
Length-for-age/height-for age *z* score, median (IQR)	−1.00 (−2.33 to 0.31)	−2.85 (−3.47 to −2.35)	−3.06 (−3.84 to −2.29)	−0.12 (−0.91 to 0.67)^b^^,^^d^	−0.36 (−1.15 to 0.47)
Weight-for-height *z* score, median (IQR)	−1.00 (−2.68 to 0.27)	−2.68 (−2.87 to −1.90)	−3.05 (−4.62 to −2.61)	0.62 (0.40 to 1.07)^b^	−0.23 (−1.06 to 0.50)^e^

Abbreviation: IQR, interquartile range.

^a^ Images refer to the number of hemotoxylin-eosin–stained biopsy images made available to the deep learning network; these included both scanned images (celiac disease and no disease) and digitized images (environmental enteropathy from Pakistan and Zambia). For Pakistan, there were 2 to 3 biopsies available from each patient; therefore, there were 29 digitized biopsy images from 10 patients.

^b^ Three patients with celiac disease did not have anthropometric data available, and they were excluded from the analysis for all *z* scores.

^c^ Weight-for-age *z *scores could only be calculated for approximately 35% of patients with celiac disease because the rest were older than 10 years and there is no reference standard for this age group.

^d^ Weight-for-height *z* scores could only be generated for 7 patients with celiac disease using the current algorithm.

^e^ Weight-for-height *z* scores could only be generated for 38 patients with no disease using the algorithm.

### Deep Learning Prediction Accuracy

We used a case-preserving 10-fold cross-validation setup, ie, all images from a given patient were either in the training set or the testing set for a given fold. The models were trained for only 20 epochs to avoid overfitting and achieved 92.1% cross-validation per-image prediction accuracy (evaluated for each image individually) and 93.4% per-patient accuracy (evaluated after taking mean probabilities from all images). Aggregated confusion/error matrices (a table representing a CNN’s predicted classification vs actual classification, enabling visualization of the algorithm’s performance^49,50^) were generated for the 10-fold cross-validation to understand where most incorrect classifications occurred, and most were found between patients with CD and the control group.^41^ On review, all misclassified CD biopsies had a Marsh score of 1. Overall, based on our aggregated confusion matrix, our model had a false-negative rate of 2.4%.

### Deconvolutional Neural Networks Paired With CNN

**Figure 2.**
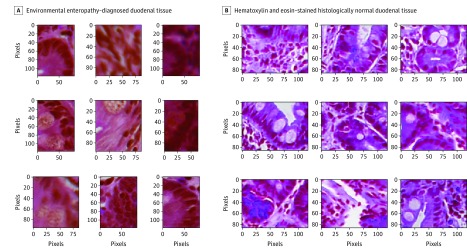
High Activation Areas A, Hematoxylin-eosin–stained duodenal tissues with diagnosed environmental enteropathy (original magnification ×100). B, Hematoxylin-eosin–stained histologically normal duodenal tissue (original magnification ×100). These images were the areas of high activation identified by the model; we observed secretory cells, specifically Paneth cells and goblet cells, in the mucosa. Our classification model identified these secretory cells to be of high importance for distinguishing biopsies with no disease from biopsies of environmental enteropathy and celiac disease.

Various DNN feature maps learned distinctive patterns, and as a result, the highest 9 activations corresponded to relatively similar segments from the training data. The model automatically learned microlevel features in the data, specifically duodenal epithelial secretory cells, which were identified as highly important in the predictive diagnosis of EE or CD (Figure 2).

**Figure 3.**
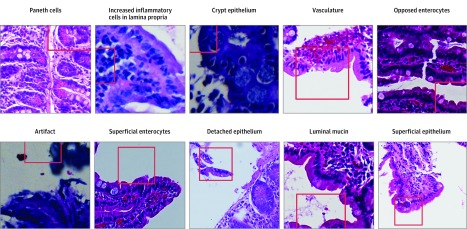
Deconvolution Groupings We selected 151 deconvolutions from hematoxylin-eosin–stained duodenal biopsies for interpretation (original magnification ×40); the 10 groupings the model identified are shown. Red boxes and lines indicate the pixel configuration that the deconvolution model considered an area of importance. Each of these features was used by the model in its decision-making process, but the relative importance of each feature is unknown.

Furthermore, we extracted 151 deconvolutions to gain insight into the model’s decision-making process. The deconvolutions generated were reviewed by a gastrointestinal pathologist (C.A.M.) and pediatric gastroenterologist (S.S.) and broadly categorized into 10 groups, including Paneth cells, luminal mucin, apposed epithelium, and artifact (Figure 3) (eAppendix 3 in the Supplement).

### Intercountry EE Comparison

Because we had EE biopsies from Zambia and Pakistan, we were interested in analyzing the intercountry microfeature differences. First, all patients from Zambia were excluded, and 10 models were trained, each of which removed 1 of the 10 patients from Pakistan. Control and CD images were randomly allocated to the models. After training, the patients from Zambia were used for evaluation. Table 2 shows the per-image and per-case performance. Overall, 3 models misclassified almost all specimens from Zambia. We identified the specimens from Pakistan that were removed from each model; therefore, these 3 specimens from Pakistan were hypothesized to be very informative for the identification of EE in the images from Zambia. To validate this, an additional model was trained using only the 3 biopsies from the 3 patients Pakistan and was tested on all patients from Zambia, achieving 99.0% per-image and 100% per-case accuracy.

**Table 2.** Classification Accuracy of Model Trained on Patients From Pakistan and Evaluated on Patients From Zambia

Evaluation Method	Model No.									
	1	2	3	4	5	6	7	8	9	10
Per-image accuracy	0.94	0.93	0.92	0.07	0.81	0	0.91	0.997	0.14	0.89
Per-case accuracy	1.00	1.00	1.00	0.13	1.00	0	1.00	1.00	0.13	1.00

### Biopsy Patterns–Biomarker Correlation Model

We found that the mean 10-fold cross-validation MSE was 0.0840, with a variance of 0.0038. The top 5 biomarkers identified were interleukin 9, interleukin 6, interleukin 1b, interferon γ-induced protein 10, and regenerating family member 1. eFigure 4 in the Supplement shows that, by using the 12 most important features, we achieved the lowest per-image error.

We qualitatively compared the 32 regions that corresponded to the highest activation values for the 32 feature maps against the regions obtained from the correlation algorithm (eFigure 5 in the Supplement). Segments were sorted by the absolute difference between the estimated value, produced by the regression model and matched to the closest corresponding training biopsy segment, and the ground-truth activation values, which corresponded to the highest 32 activations from each EE specimen in the testing set. Our correlation algorithm produced relatively good estimates (eFigure 5 in the Supplement), and the MSE was 0.0751. However, given the minimal biomarker overlap between specimens from Pakistan and Zambia, this proposed method needs to be validated in a larger data set before biological interpretation of the meaning of the correlations can be attempted.

## Discussion

This study aimed to develop and validate a machine learning–based histopathological analysis model to distinguish and extract morphologic phenotypes from duodenal biopsy images and identify novel features that could be used to help differentiate between overlapping conditions causing pediatric undernutrition. The major results of this work include the following: (1) the development of a CNN applied to H-E–stained duodenal biopsy specimens from participants with healthy tissue, CD, and EE; (2) the use of a DNN to identify distinguishing features; and (3) a proposed analytic framework to correlate high-dimensional biomarker data with biopsy features.

In the past decade, various studies have investigated the use of deep learning to facilitate the detection of medical conditions.^1,51,52,53,54,55,56,57,58,59^ In 2017, Ehteshami Bejnordi et al^1^ used deep learning algorithms to interpret sentinel lymph node pathology images. They used whole-slide images, which had been annotated for metastases, as their input to train the algorithms.^1^ Our analytic framework was set up as a cross-validation, ie, our training set was labeled but our validation set was not. We did not apply any annotations other than the assignment of broad categories (ie, EE, CD, or control). Strengths of our study include a novel machine learning–based histopathological analysis for identifying and differentiating between gastrointestinal diseases and control images and the use of a DNN for feature recognition and novel insights into differentiating 2 histologically similar diseases.

### Limitations

This study has some inherent limitations. First, we had different forms of images as inputs, including (1) digitized slides for EE (from Pakistan and Zambia) and (2) images taken from a microscope at different resolutions for CD and the control group. Therefore, the data from our patients with EE were much larger in size and more feature heavy. Second, there was an obvious intersite staining color difference, leading to a potential decision-making bias based on color and likely producing our high degree of accuracy. Although H-E staining is a standard method used by pathologists to study human tissue, differences in commercially available reagents by country led to clear color differences in biopsy slides from Zambia, Pakistan, and the United States. We used γ correction to address this problem, but this only partly solved the issue, evident by the high accuracy of EE classification. Nevertheless, in the context of the scarce literature about applying CNNs to small-intestinal tissue, our results suggest that CNNs can be used to provide quantitative and novel disease insights. Third, our study had broad inclusion criteria for the control group. While pathology reports confirmed healthy small-intestine tissue, patients were not excluded from the study on the basis of disease in other parts of the gastrointestinal tract (eg, eosinophilic esophagitis). Our current work includes more stringent inclusion and exclusion criterion for both CD and the control group. We presented a proposed analytic framework for biopsy pattern correlation with biomarkers; however, our small data set limited us from making biological inferences regarding the biopsy feature groupings identified via biomarkers. Future directions for our research include using digitized images for all disease categories, thereby providing the algorithm with high-resolution microscopic features, hypothetically enabling it to more robustly identify novel features and decreasing the amount of artifact used for decision making as data size increases. Future work will also include transfer-learning approaches, ideally allowing the algorithm to make more accurate classifications and feature predictions. Gradient-weighted class activation mapping^60^ will be applied for the assignment of relative-importance weights to distinguishing features on each image and disease. Additionally, to address differential staining between study sites and reduce appearance variability within the data set, methods of stain normalization will be implemented, which will modify the image color to resemble a reference sample. Furthermore, a potential reason for most misclassifications occurring between CD and healthy tissue could be that the misclassified CD images had a lower Marsh score or unusual clinical features (eg, normal serology, no weight loss, constipation vs diarrhea). We plan to conduct a secondary review of these misclassifications to identify histological and clinical features that could have caused misclassifications. Further, we plan to expand our current biomarker analysis to correlate microscopic biopsy features with a wide array of biomarker as well as molecular and genetic data.

## Conclusions

In this diagnostic study, a machine learning–based histopathological analysis model demonstrated 93.4% classification accuracy for identifying and differentiating between duodenal biopsies from children with EE and CD. The combination of CNNs with a DNN enabled feature recognition and highlighted secretory cells’ role in the model’s ability to differentiate between these histologically similar diseases.

## Notes

Supplement. **eAppendix 1. **Supplemental Methods **eAppendix 2.** Supplemental Results **eAppendix 3. **Supplemental Discussion **eFigure 1. **Methods of Artificial Data Augmentation **eFigure 2. **Feature Predictions *t *Tests **eFigure 3. **An Illustration of Biopsy Features Correlation Framework Using Data From Pakistan **eFigure 4. **Importance of Noninvasive Biomarkers Estimated Using Random Forest Model Using Data From Pakistan **eFigure 5. **A Comparison Between 32 Biopsy Segments Generated From Correlation Algorithm and Ground-Truth Obtained From a Testing Case Using Data From Pakistan **eReferences**

## References

[1] Ehteshami Bejnordi B, Veta M, Johannes van Diest P, ; CAMELYON16 Consortium Diagnostic assessment of deep learning algorithms for detection of lymph node metastases in women with breast cancer. JAMA. 2017;318(22):-. doi:10.1001/jama.2017.14585 29234806 PMC5820737

[2] LeCun Y, Bengio Y, Hinton G Deep learning. Nature. 2015;521(7553):436-444. doi:10.1038/nature14539 26017442

[3] Golden JA Deep learning algorithms for detection of lymph node metastases from breast cancer: helping artificial intelligence be seen. JAMA. 2017;318(22):2184-2186. doi:10.1001/jama.2017.14580 29234791

[4] Beam AL, Kohane IS Big data and machine learning in health care. JAMA. 2018;319(13):1317-1318. doi:10.1001/jama.2017.18391 29532063

[5] World Health Organization Children: reducing mortality. https://www.who.int/en/news-room/fact-sheets/detail/children-reducing-mortality. Accessed April 9, 2018.

[6] World Health Organization Joint child malnutrition estimate: levels and trends (2017 edition). https://www.who.int/nutgrowthdb/estimates2016/en/. Accessed April 9, 2018.

[7] Arndt MB, Richardson BA, Ahmed T, ; MAL-ED Network Project Fecal markers of environmental enteropathy and subsequent growth in Bangladeshi children. Am J Trop Med Hyg. 2016;95(3):694-701. doi:10.4269/ajtmh.16-0098 27352872 PMC5014281

[8] Guerrant RL, Leite AM, Pinkerton R, Biomarkers of environmental enteropathy, inflammation, stunting, and impaired growth in children in northeast Brazil. PLoS One. 2016;11(9):e0158772. doi:10.1371/journal.pone.0158772 27690129 PMC5045163

[9] Korpe PS, Petri WA Jr Environmental enteropathy: critical implications of a poorly understood condition. Trends Mol Med. 2012;18(6):328-336. doi:10.1016/j.molmed.2012.04.007 22633998 PMC3372657

[10] Naylor C, Lu M, Haque R, ; PROVIDE study teams Environmental enteropathy, oral vaccine failure and growth faltering in infants in Bangladesh. EBioMedicine. 2015;2(11):1759-1766. doi:10.1016/j.ebiom.2015.09.036 26870801 PMC4740306

[11] Rubio-Tapia A, Ludvigsson JF, Brantner TL, Murray JA, Everhart JE The prevalence of celiac disease in the United States. Am J Gastroenterol. 2012;107(10):1538-1544. 22850429 10.1038/ajg.2012.219

[12] Husby S, Koletzko S, Korponay-Szabó IR, ; ESPGHAN Working Group on Coeliac Disease Diagnosis; ESPGHAN Gastroenterology Committee; European Society for Pediatric Gastroenterology, Hepatology, and Nutrition European Society for Pediatric Gastroenterology, Hepatology, and Nutrition guidelines for the diagnosis of coeliac disease. J Pediatr Gastroenterol Nutr. 2012;54(1):136-160. doi:10.1097/MPG.0b013e31821a23d0 22197856

[13] Ali A, Iqbal NT, Sadiq K Environmental enteropathy. Curr Opin Gastroenterol. 2016;32(1):12-17. doi:10.1097/MOG.0000000000000226 26574871

[14] Uddin MI, Islam S, Nishat NS, Biomarkers of environmental enteropathy are positively associated with immune responses to an oral cholera vaccine in Bangladeshi children. PLoS Negl Trop Dis. 2016;10(11):e0005039. doi:10.1371/journal.pntd.0005039 27824883 PMC5100882

[15] Campbell DI, Elia M, Lunn PG Growth faltering in rural Gambian infants is associated with impaired small intestinal barrier function, leading to endotoxemia and systemic inflammation. J Nutr. 2003;133(5):1332-1338. doi:10.1093/jn/133.5.1332 12730419

[16] Solomons NW Environmental contamination and chronic inflammation influence human growth potential. J Nutr. 2003;133(5):1237. doi:10.1093/jn/133.5.1237 12730402

[17] Syed S, Ali A, Duggan C Environmental enteric dysfunction in children. J Pediatr Gastroenterol Nutr. 2016;63(1):6-14. doi:10.1097/MPG.0000000000001147 26974416 PMC4920693

[18] Syed S, Dinallo V, Iqbal NT, High SMAD7 and p-SMAD2,3 expression is associated with environmental enteropathy in children. PLoS Negl Trop Dis. 2018;12(2):e0006224. doi:10.1371/journal.pntd.0006224 29415065 PMC5819826

[19] Syed S, Yeruva S, Herrmann J, Environmental enteropathy in undernourished Pakistani children: clinical and histomorphometric analyses. Am J Trop Med Hyg. 2018;98(6):1577-1584. doi:10.4269/ajtmh.17-0306 29611507 PMC6086170

[20] Ramakrishna BS, Venkataraman S, Mukhopadhya A Tropical malabsorption. Postgrad Med J. 2006;82(974):779-787. doi:10.1136/pgmj.2006.048579 17148698 PMC2653921

[21] Sullivan PB, Marsh MN, Mirakian R, Hill SM, Milla PJ, Neale G Chronic diarrhea and malnutrition: histology of the small intestinal lesion. J Pediatr Gastroenterol Nutr. 1991;12(2):195-203. doi:10.1097/00005176-199102000-00010 1904932

[22] Rubio-Tapia A, Hill ID, Kelly CP, Calderwood AH, Murray JA; American College of Gastroenterology ACG clinical guidelines: diagnosis and management of celiac disease. Am J Gastroenterol. 2013;108(5):656-676. doi:10.1038/ajg.2013.79 23609613 PMC3706994

[23] Green PH, Cellier C Celiac disease. N Engl J Med. 2007;357(17):1731-1743. doi:10.1056/NEJMra071600 17960014

[24] Sharma G, Carter A Artificial intelligence and the pathologist: future frenemies? Arch Pathol Lab Med. 2017;141(5):622-623. doi:10.5858/arpa.2016-0593-ED 28447905

[25] Gurcan MN, Boucheron LE, Can A, Madabhushi A, Rajpoot NM, Yener B Histopathological image analysis: a review. IEEE Rev Biomed Eng. 2009;2:147-171. doi:10.1109/RBME.2009.2034865 20671804 PMC2910932

[26] Corazza GR, Villanacci V, Zambelli C, Comparison of the interobserver reproducibility with different histologic criteria used in celiac disease. Clin Gastroenterol Hepatol. 2007;5(7):838-843. doi:10.1016/j.cgh.2007.03.019 17544877

[27] Mubarak A, Nikkels P, Houwen R, Ten Kate F Reproducibility of the histological diagnosis of celiac disease. Scand J Gastroenterol. 2011;46(9):1065-1073. doi:10.3109/00365521.2011.589471 21668407

[28] West D Eliminating subjectivity in pathology. https://www.technologynetworks.com/diagnostics/articles/eliminating-subjectivity-in-pathology-292836. Accessed May 8, 2019.

[29] Zeiler MD, Fergus R Visualizing and understanding convolutional networks. https://arxiv.org/abs/1311.2901. Accessed May 13, 2019.

[30] Zeiler MD, Krishnan D, Taylor GW, Fergus R Deconvolutional networks In: 2010 IEEE Computer Society Conference on Computer Vision and Pattern Recognition. Piscataway, NJ: Institute of Electrical and Electronics Engineers; 2010:2528-2535.

[31] Collins GS, Reitsma JB, Altman DG, Moons KG Transparent reporting of a multivariable prediction model for individual prognosis or diagnosis (TRIPOD): the TRIPOD statement. BMJ. 2015;350:g7594. doi:10.1136/bmj.g7594 25569120

[32] Iqbal NT, Sadiq K, Syed S, Promising biomarkers of environmental enteric dysfunction: a prospective cohort study in Pakistani children. Sci Rep. 2018;8(1):2966. doi:10.1038/s41598-018-21319-8 29445110 PMC5813024

[33] Amadi B, Besa E, Zyambo K, Impaired barrier function and autoantibody generation in malnutrition enteropathy in Zambia. EBioMedicine. 2017;22:191-199. doi:10.1016/j.ebiom.2017.07.017 28750860 PMC5552244

[34] World Health Organization WHO Antrho (version 3.2.2, January 2011) and macros. https://www.who.int/childgrowth/software/en/. Accessed May 8, 2019.

[35] World Health Organization Growth reference data for 5-19 years. https://www.who.int/growthref/en/. Accessed May 8, 2019.

[36] Krizhevsky A, Sutskever I, Hinton GE Imagenet classification with deep convolutional neural networks In: Pereira F, Burges CJC, Bottou L, Weinberger KQ, eds. Advances in Neural Information Processing Systems. San Diego, CA: Neural Information Processing Systems, Inc; 2012:1097-1105.

[37] Simonyan K, Zisserman A Very deep convolutional networks for large-scale image recognition. https://arxiv.org/abs/1409.1556. Accessed May 8, 2019.

[38] He K, Zhang X, Ren S, Sun J Deep residual learning for image recognition In: 2016 IEEE Conference on Computer Vision and Pattern Recognition. Piscataway, NJ: Institute of Electrical and Electronics Engineers; 2016:770-778.

[39] Nair V, Hinton GE Rectified linear units improve restricted Boltzmann machines In: Fürnkranz J, Joachims T, eds. Proceedings of the 27th International Conference on International Conference on Machine Learning. Madison, WI: Omnipress; 2010:807-814.

[40] Hinton GE, Srivastava N, Krizhevsky A, Sutskever I, Salakhutdinov RR Improving neural networks by preventing co-adaptation of feature detectors. https://arxiv.org/abs/1207.0580. Accessed May 8, 2019.

[41] Al-Boni M, Syed S, Ali A, Moore S, Brown DE Duodenal biopsies classification and understanding using convolutional neural networks In: AMIA Informatics Summit Proceedings. Bethesda, MA: American Medical Informatics Association; 2019. PMC6568096 31258999

[42] Harper KM, Mutasa M, Prendergast AJ, Humphrey J, Manges AR Environmental enteric dysfunction pathways and child stunting: a systematic review. PLoS Negl Trop Dis. 2018;12(1):e0006205. doi:10.1371/journal.pntd.0006205 29351288 PMC5792022

[43] Mahfuz M, Das S, Mazumder RN, Bangladesh Environmental Enteric Dysfunction (BEED) study: protocol for a community-based intervention study to validate non-invasive biomarkers of environmental enteric dysfunction. BMJ Open. 2017;7(8):e017768. doi:10.1136/bmjopen-2017-017768 28801442 PMC5724211

[44] Campbell RK, Schulze KJ, Shaikh S, Biomarkers of environmental enteric dysfunction among children in rural Bangladesh. J Pediatr Gastroenterol Nutr. 2017;65(1):40-46. doi:10.1097/MPG.0000000000001557 28644348 PMC5492885

[45] Locks LM, Mwiru RS, Mtisi E, Infant nutritional status and markers of environmental enteric dysfunction are associated with midchildhood anthropometry and blood pressure in Tanzania. J Pediatr. 2017;187:225-233.e1. 28499715 10.1016/j.jpeds.2017.04.005 PMC5533170

[46] Lertudomphonwanit C, Mourya R, Fei L, Large-scale proteomics identifies MMP-7 as a sentinel of epithelial injury and of biliary atresia. Sci Transl Med. 2017;9(417):eaan8462. 29167395 10.1126/scitranslmed.aan8462 PMC5902315

[47] Boden EK, Shows DM, Chiorean MV, Lord JD Identification of candidate biomarkers associated with response to vedolizumab in inflammatory bowel disease. Dig Dis Sci. 2018;63(9):2419-2429. doi:10.1007/s10620-018-4924-8 29372476

[48] Morris MW, Stewart SA, Heisler C, Biomarker-based models outperform patient-reported scores in predicting endoscopic inflammatory disease activity. Inflamm Bowel Dis. 2018;24(2):277-285. doi:10.1093/ibd/izx018 29361090

[49] Powers DM Evaluation: from precision, recall and F-measure to ROC, informedness, markedness and correlation. https://dspace.flinders.edu.au/xmlui/handle/2328/27165. Accessed May 8, 2019.

[50] Stehman SV Selecting and interpreting measures of thematic classification accuracy. Remote Sens Environ. 1997;62(1):77-89. doi:10.1016/S0034-4257(97)00083-7

[51] Gargeya R, Leng T Automated identification of diabetic retinopathy using deep learning. Ophthalmology. 2017;124(7):962-969. doi:10.1016/j.ophtha.2017.02.008 28359545

[52] Ting DSW, Cheung CY, Lim G, Development and validation of a deep learning system for diabetic retinopathy and related eye diseases using retinal images from multiethnic populations with diabetes. JAMA. 2017;318(22):2211-2223. doi:10.1001/jama.2017.18152 29234807 PMC5820739

[53] Gulshan V, Peng L, Coram M, Development and validation of a deep learning algorithm for detection of diabetic retinopathy in retinal fundus photographs. JAMA. 2016;316(22):2402-2410. doi:10.1001/jama.2016.17216 27898976

[54] Abràmoff MD, Lou Y, Erginay A, Improved automated detection of diabetic retinopathy on a publicly available dataset through integration of deep learning. Invest Ophthalmol Vis Sci. 2016;57(13):5200-5206. doi:10.1167/iovs.16-19964 27701631

[55] Becker AS, Marcon M, Ghafoor S, Wurnig MC, Frauenfelder T, Boss A Deep learning in mammography: diagnostic accuracy of a multipurpose image analysis software in the detection of breast cancer. Invest Radiol. 2017;52(7):434-440. doi:10.1097/RLI.0000000000000358 28212138

[56] Bejnordi BE, Lin J, Glass B, Deep learning-based assessment of tumor-associated stroma for diagnosing breast cancer in histopathology images In: 2017 IEEE 14th International Symposium on Biomedical Imaging. Piscataway, NJ: Institute of Electrical and Electronics Engineers; 2017:929-932. 10.1109/ISBI.2017.7950668 PMC6802272 31636811

[57] Cruz-Roa A, Basavanhally A, González F, Automatic detection of invasive ductal carcinoma in whole slide images with convolutional neural networks In: Gurcan MN, Madabhushi A, eds. Medical Imaging 2014: Digital Pathology. Bellingham, WA: The International Society for Optics and Photonics; 2014:904103.

[58] Wang D, Khosla A, Gargeya R, Irshad H, Beck AH Deep learning for identifying metastatic breast cancer. https://arxiv.org/abs/1606.05718. Accessed May 8, 2019.

[59] Beck AH, Sangoi AR, Leung S, Systematic analysis of breast cancer morphology uncovers stromal features associated with survival. Sci Transl Med. 2011;3(108):108ra113. doi:10.1126/scitranslmed.3002564 22072638

[60] Selvaraju RR, Cogswell M, Das A, Vedantam R, Parikh D, Batra D Grad-CAM: visual explanations from deep networks via gradient-based localization. https://arxiv.org/abs/1610.02391. Accessed May 8, 2019.

