# Error in Funding/Support

**DOI:** 10.1001/jamanetworkopen.2019.7967

**Published:** 2019-07-03

In the Original Investigation titled “Assessment of Machine Learning Detection of Environmental Enteropathy and Celiac Disease in Children,”^1^ published June 14, 2019, there was an error in the Funding/Support section of the Article Information. It should have read, “This work was supported by the University of Virginia Center for Engineering in Medicine Grant awarded to Drs Syed and Brown, grant OPP1066203 from the Bill and Melinda Gates Foundation awarded to Dr Ali, grant OPP1066118 from the Bill and Melinda Gates Foundation awarded to Dr Kelly, and the University of Virginia THRIV Scholar Career Development Award awarded to Dr Syed.” This article has been corrected.^1^

## References

[1] Syed S, Al-Boni M, Khan MN, Assessment of machine learning detection of environmental enteropathy and celiac disease in children. JAMA Netw Open. 2019;2(6):. doi:10.1001/jamanetworkopen.2019.5822 10.1001/jamanetworkopen.2019.5822 PMC6575155 31199451

