# Supplementary material for: Assessment of Machine Learning Detection of Environmental Enteropathy and Celiac Disease in Children
Source: JAMA Netw Open. 2019 Jun 14;2(6):e195822. doi: 10.1001/jamanetworkopen.2019.5822 (PMC6575155; doi:10.1001/jamanetworkopen.2019.5822)
Supplement: Supplement. — eAppendix 1. Supplemental Methods eAppendix 2. Supplemental Results eAppendix 3. Supplemental Discussion eFigure 1. Methods of Artificial Data Augmentation eFigure 2. Feature Predictions t Tests eFigure 3. An Illustration of Biopsy Features Correlation Framework Using Data From Pakistan eFigure 4. Importance of Noninvasive Biomarkers Estimated Using Random Forest Model Using Data From Pakistan eFigure 5. A Comparison Between 32 Biopsy Segments Generated From Correlation Algorithm and Ground-Truth Obtained From a Testing Case Using Data From Pakistan eReferences [file jamanetwopen-2-e195822-s001.pdf]

Supplementary Online Content

Syed S, Al-Boni M, Khan MN, et al. Assessment of machine learning detection of environmental enteropathy and celiac disease in children. *JAMA Netw Open*. 2019;2(6):e195822. doi:10.1001/jamanetworkopen.2019.5822

eAppendix 1. Supplemental Methods

eAppendix 2. Supplemental Results

eAppendix 3. Supplemental Discussion

eFigure 1. Methods of Artificial Data Augmentation

eFigure 2. Feature Predictions *t* Tests

eFigure 3. An Illustration of Biopsy Features Correlation Framework Using Data From Pakistan

eFigure 4. Importance of Noninvasive Biomarkers Estimated Using Random Forest Model Using Data From Pakistan

eFigure 5. A Comparison Between 32 Biopsy Segments Generated From Correlation Algorithm and Ground-Truth Obtained From a Testing Case Using Data From Pakistan

eReferences

This supplementary material has been provided by the authors to give readers additional information about their work.

© 2019 Syed S et al. *JAMA Network Open.*

eAppendix 1. Supplemental Methods

Patient selection

Celiac Disease (CD) and histologically normal control populations were selected from participants who had undergone esophagogastroduodenoscopy (EGD) and biopsy at UVa in the past 20 years. Our criteria for participant sample selection included: 1) age < 6 years for histologically normal controls, and age < 18 years for CD diagnoses, 2) the histopathology report confirmed a diagnosis of CD or reported normal small intestinal biopsy findings, and 3) the corresponding H&E stained slide from the procedure was available at the University of Virginia Biorepository and Tissue Research Facility (BTRF). 76 participants (CD = 34, histologically normal = 42) met our selection criteria and each slide represented all the duodenal samples collected during one EGD.

Participants with EE from Pakistan were part of a larger study that investigated novel biomarkers of EE[1]. This was a prospective birth cohort (n = 380 children) in the rural district of Matiari, Pakistan, where children were followed monthly for growth faltering[1]. Those with a clinical concern for malabsorptive organic pathology (with symptoms such as persistent diarrhea, bloating, etc.) were evaluated by a clinical pediatric gastroenterologist, and diagnostic endoscopies were performed on 11 children. The study case definition of EE included any child with wasting (weight-for-age Z score ≤ -2) found to have weight and height refractory to nutritional intervention[1]. 10 children were found to have EE[1,2], with 2-3 duodenal biopsies per child for a total of 29 digitized duodenal biopsies available from this subset.

Participants with EE from Zambia were part of another study conducted at the University Teaching Hospital, Lusaka that selected children with severe acute malnutrition[3]. The parent study was designed to investigate the morphological changes and barrier function of the small intestine in children with malnutrition[3]. 34 children with severe acute malnutrition (defined as low weight for height (wasting), i.e. weight-for-height Z score < -2) with persistent diarrhea in whom no cause was identified were hospitalized for endoscopic evaluation[3,4], any children who were considered too ill for the procedure or had significant congenital abnormalities were excluded [3,4]. Of the children undergoing endoscopy, 16 children were found to have abnormal duodenal histology and were diagnosed with EE. We had access to a single digitized slide per child with EE with each slide having a single duodenal biopsy piece (except for 3/16 cases where there were 2 biopsy pieces) per glass slide.

© 2019 Syed S et al. *JAMA Network Open.*

eAppendix 2. Supplemental Results

Background clinical characteristics of patient populations

The characteristics of the EE patients have already been published [2,3], to summarize the EE patients from Pakistan had a median (Q1, Q3) age of 22 (20, 23) months, 50% were female, 50% (n = 5) had low birth weight (<2500 g), and 60% (n =6) were born premature (< 37 weeks of gestation) [2]. The EE patients from Zambia had a median (Q1, Q3) age of 16.5 (10.5, 21) months, 37.5% were female, and 31.25% were HIV positive. Further details about the characteristics of the patients from Zambia are described by Amadi et al [3].

Of the 34 CD patients and 42 histologically normal controls, 65% and 38% were female, respectively. The ages of the subsets varied, where the median (Q1, Q3) age for the CD group was 129 (72.5, 180.75) months, and that for the histologically normal control group was 31.5 (22, 49.75) months. LAZ scores could not be calculated for 3 of the CD patients due to missing data, and WAZ scores could only be calculated on 12 of the CD patients as the rest were over the age of 10 years. Of the 31 CD patients, 3 were found to have LAZ < -2. Z scores could be calculated for all of the 42 histologically normal control patients, 5 were found to have LAZ < -2, and 5 were found to have WAZ < -2. Overall, the majority of our patient population from these two groups had appropriate weight and height according to their age.

© 2019 Syed S et al. *JAMA Network Open.*

eAppendix 3. Supplemental Discussion

Deconvolution based categories

Paneth cells play an integral role in our innate immunity, and have been shown to decrease in number in complicated cases of celiac disease [5]. While we were not able to quantify the increase or decrease in Paneth cells per disease category, the deconvolutions predict that they were one of the features used for classification. The deconvolutions also showed detached epithelium and luminal mucin, both of which could be linked to the loss of the epithelial glycocalyx. The glycocalyx is a meshwork formed by glycolipids and/or glycoproteins and protects the intestine by forming a barrier against invading bacteria at the epithelial cell surface [6]. The loss of glycocalyx has previously been observed to be found in the small intestinal biopsies from children with chronic diarrhea and an intolerance to dietary proteins, and it was hypothesized that this may be an immunologic process and not a direct host-protein interaction [7]. EE has commonly been associated with altered barrier function [1,8,9] and we hypothesize that further studying the loss of the glycocalyx could aid in the understanding of the pathophysiology of EE. Further, the detached enterocytes could also serve as a measure of the degree of tissue edema or inflammation. Another interesting group within our deconvolutions was that of “apposed epithelium or enterocytes” – on discussion, we thought this was representative of normal villus architecture, where immediately-adjacent villi would feature apposed epithelial surfaces, while such a feature would be lost in villus atrophy. Both celiac disease [10,11] and EE [2,8,9,12] are known to be associated with villus atrophy, and these convolutions likely indicate that villus structure, specifically villus length and the degree of atrophy, was an important factor for classification.

© 2019 Syed S et al. *JAMA Network Open.*

eFigure 1. Methods of Artificial Data Augmentation

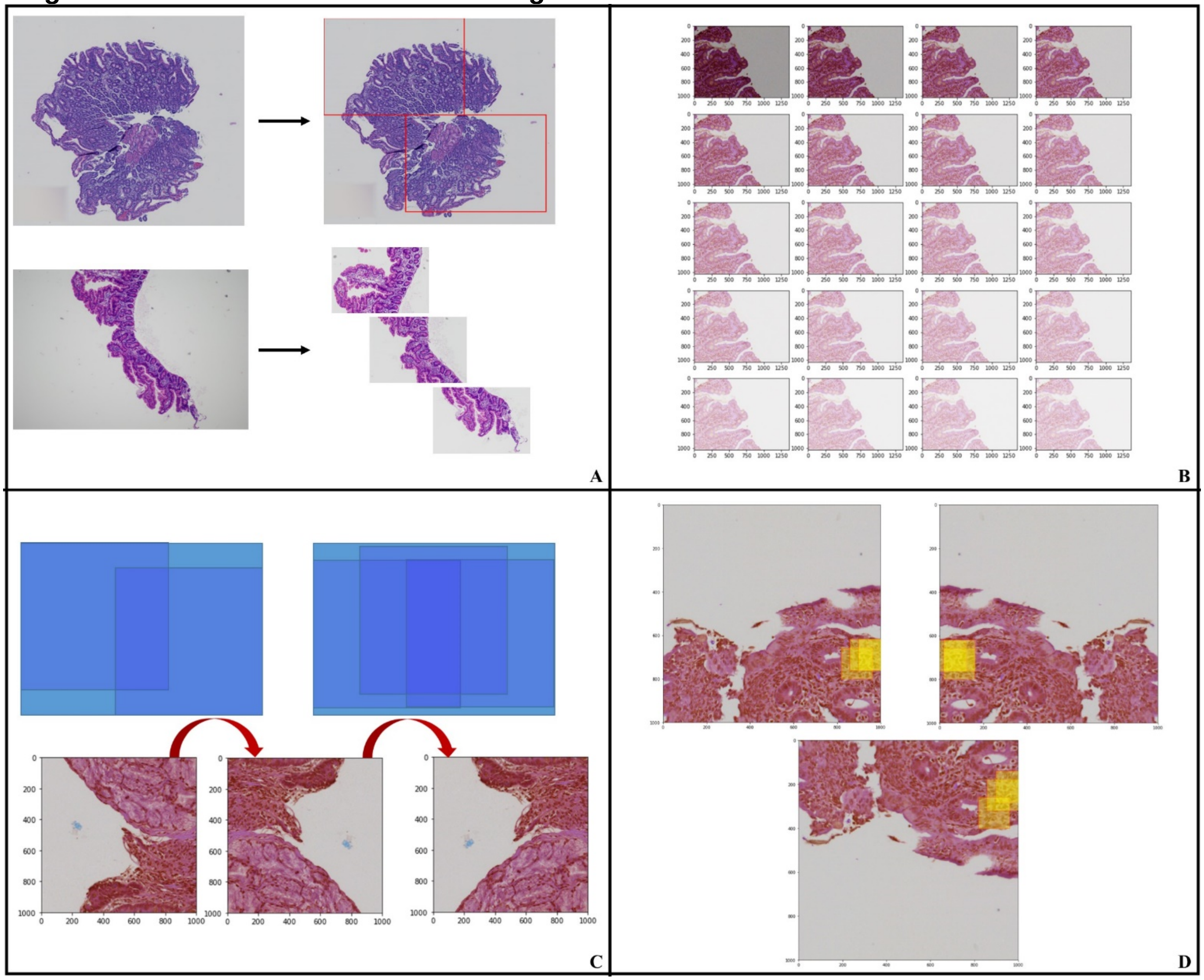

We augmented our data by segmentation, horizontal and vertical reflection of randomly selected patches, and gamma correction. A) Biopsy segmentation; the top image shows automatic segmentation (red boxes), and the bottom image shows manual segmentation; B) Gamma correction; as the biopsy slides were from different centers around the world the H&E stain varied slightly – gamma correction was done to ensure that all the images fed to the deep learning net would not be differentiated on the basis of color but truly on the basis of features present; C) Random selection of patches (blue boxes) and their horizontal and vertical reflections; D) Translation and rotation invariant features; even in different orientations the activations (yellow boxes) corresponded to the same features on each image.

© 2019 Syed S et al. *JAMA Network Open.*

eFigure 2. Feature Predictions *t* Tests

Label	μ_{29}
EE	0.2040
C	0.3130
N	**1.3455**

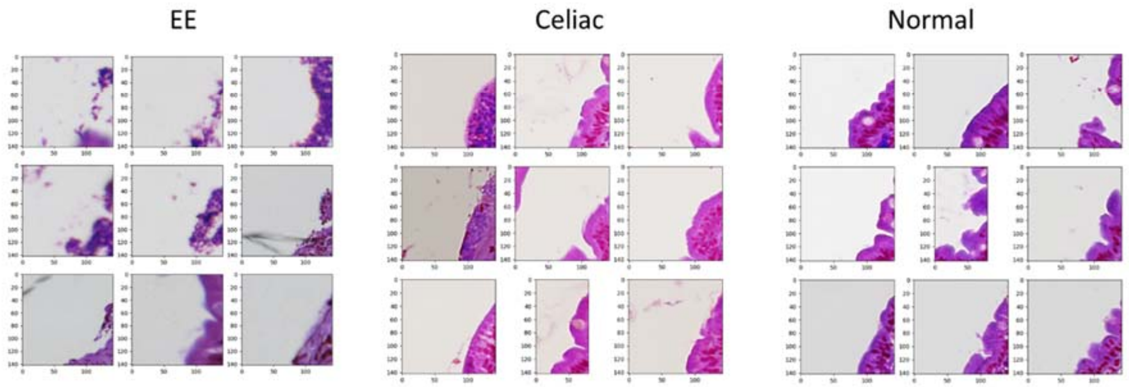

The activation T-tests serve as a proxy for testing the prevalence of various tissue patterns in different biopsies. In the shown images we are looking at Feature Map 29 which indicates a high activation feature in normal versus celiac and EE (Environmental Enteropathy). This could be a higher presence of goblet cells in normal tissue when compared to celiac and EE or epithelial cell configuration relative to inflammatory cells

eFigure 3. An Illustration of Biopsy Features Correlation Framework Using Data From Pakistan

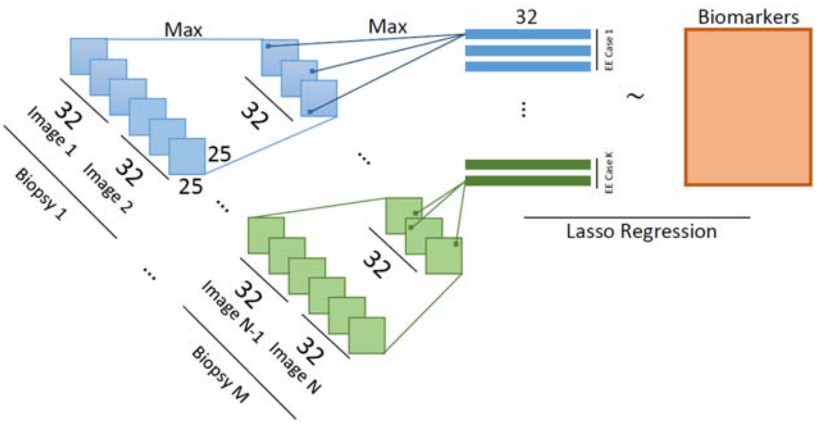

Our correlation frame works consisted of CNN activations and lasso regression models. Each biopsy was divided, either manually or automatically, into multiple training images which were fed into our CNN model which contained 32 different feature mapping at the fourth layer with each feature map searching for a specific pixel pattern (i.e. mucosal morphological feature) in the input images. Each image, when applied to the CNN framework, produced 25 x 25 convoluted values for each of the 32 feature maps. We then performed a maximum operator on activations across all images per each biopsy. The maximum value of the final 625 convoluted values corresponded to a 142 x 142 segment which maximally activated the feature map. Therefore, each biopsy was mapped onto 32 values that corresponded to 32 segments. We then extracted thirty two 142 x 142 image segments from each biopsy using lasso regression models and correlated their convoluted values at layer 4 with biomarkers. Abbreviations: CNN=Convolutional Neural Network; Max= maximum value.

© 2019 Syed S et al. *JAMA Network Open.*

eFigure 4. Importance of Noninvasive Biomarkers Estimated Using Random Forest Model Using Data From Pakistan

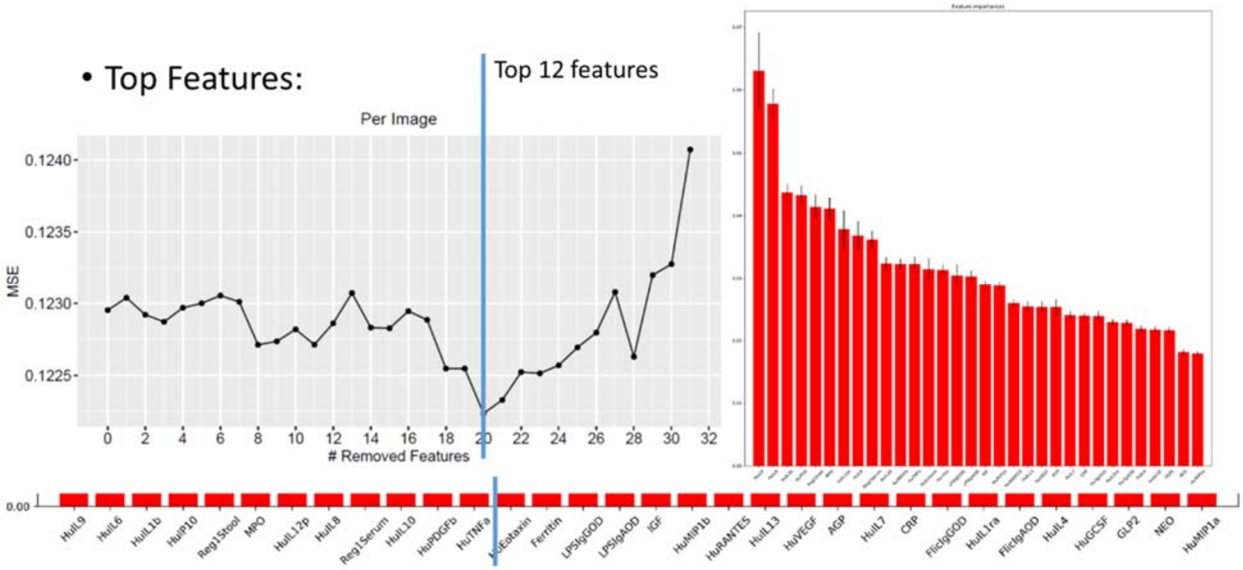

A model with a sub-set of the top 12 most important biomarkers (on the right) achieved the lowest MSE error in predicting Environmental Enteropathy biopsy patterns. Please note: Hu = Human; IL = Interleukin; Reg1 = Regenerating islet derived 1; MPO = Myeloperoxidase; PDGFb = Platelet-Derived Growth Factor Subunit B; TNFα = Tumor Necrosis Factor alpha; LPSIgG = Lipopolysaccharide Immunoglobulin G; LPSIgA = Lipopolysaccharide Immunoglobulin A; IGF = Insulin-like Growth Factor; MIP1β = Macrophage Inflammatory Protein 1-beta; RANTES = Regulated on Activation, Normal T Cell Expressed and Secreted; VEGF = Vascular endothelial growth factor; AGP = α1-acid glycoprotein; CRP = C-Reactive Protein; FlicIgG = Flagellin IgG; IL1ra = Interleukin 1 receptor antagonist; GCSF = Granulocyte Colony Stimulating Factor; GLP2 = Glucagon-like Peptide 2; NEO = neopterin; MIP1α = Macrophage Inflammatory Protein 1-alpha; MSE= mean squared error.

© 2019 Syed S et al. *JAMA Network Open.*

eFigure 5. A Comparison Between 32 Biopsy Segments Generated From Correlation Algorithm and Ground-Truth Obtained From a Testing Case Using Data From Pakistan

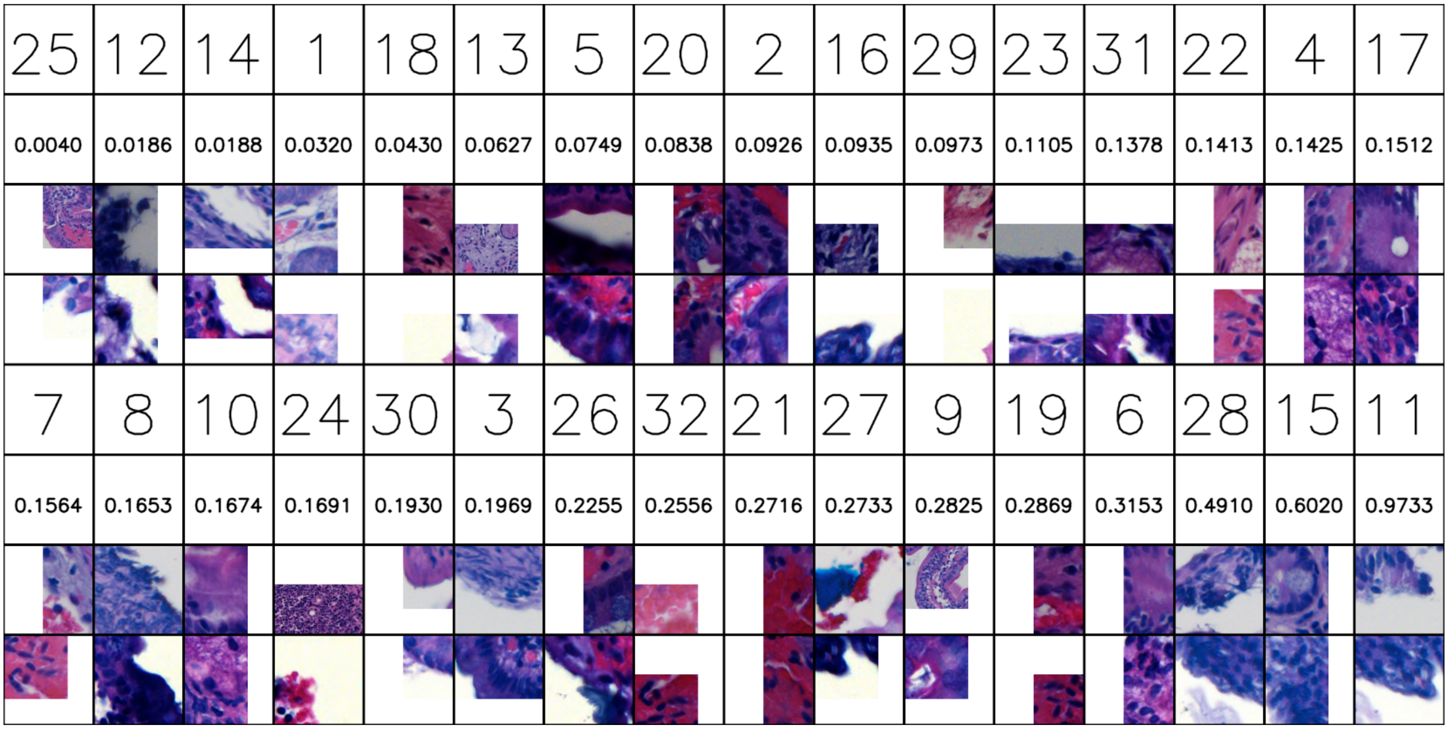

Rows 1 and 5 include the filter index. Rows 3 and 7 show the generated segments from training data. Rows 4 and 8 show the actual segments from the testing case. Rows 2 and 6 include the absolute difference between the activation values in rows 3 and 4, 7 and 8 respectively. Note, since high activations can be traced back to boundary segments, its actual size can be less than 142 x 142 pixels.

© 2019 Syed S et al. *JAMA Network Open.*

eReferences

1. Iqbal NT, Sadiq K, Syed S, et al. Promising Biomarkers of Environmental Enteric Dysfunction: A Prospective Cohort study in Pakistani Children. *Sci Rep.* 2018;8(1):2966.
2. Syed S, Yeruva S, Herrmann J, et al. Environmental Enteropathy in Undernourished Pakistani Children: Clinical and Histomorphometric Analyses. *Am J Trop Med Hyg.* 2018.
3. Amadi B, Besa E, Zyambo K, et al. Impaired Barrier Function and Autoantibody Generation in Malnutrition Enteropathy in Zambia. *EBioMedicine.* 2017;22:191-199.
4. Syed S, Dinallo V, Iqbal NT, et al. High SMAD7 and p-SMAD2,3 expression is associated with environmental enteropathy in children. *PLoS Negl Trop Dis.* 2018;12(2):e0006224.
5. Di Sabatino A, Miceli E, Dhaliwal W, et al. Distribution, proliferation, and function of Paneth cells in uncomplicated and complicated adult celiac disease. *Am J Clin Pathol.* 2008;130(1):34-42.
6. Okumura R, Takeda K. Roles of intestinal epithelial cells in the maintenance of gut homeostasis. *Experimental &Amp; Molecular Medicine.* 2017;49:e338.
7. Poley JR. Loss of the glycocalyx of enterocytes in small intestine: a feature detected by scanning electron microscopy in children with gastrointestinal intolerance to dietary protein. *J Pediatr Gastroenterol Nutr.* 1988;7(3):386-394.
8. Syed S, Ali A, Duggan C. Environmental Enteric Dysfunction in Children. *J Pediatr Gastroenterol Nutr.* 2016;63(1):6-14.
9. Harper KM, Mutasa M, Prendergast AJ, Humphrey J, Manges AR. Environmental enteric dysfunction pathways and child stunting: A systematic review. *PLoS neglected tropical diseases.* 2018;12(1):e0006205.
10. Green PH, Cellier C. Celiac disease. *N Engl J Med.* 2007;357(17):1731-1743.
11. Gujral N, Freeman HJ, Thomson AB. Celiac disease: prevalence, diagnosis, pathogenesis and treatment. *World J Gastroenterol.* 2012;18(42):6036-6059.
12. Ali A, Iqbal NT, Sadiq K. Environmental enteropathy. *Curr Opin Gastroenterol.* 2016;32(1):12-17.

© 2019 Syed S et al. *JAMA Network Open.*
